# BET inhibitors as novel therapeutic agents in breast cancer

**DOI:** 10.18632/oncotarget.19744

**Published:** 2017-08-01

**Authors:** Alberto Ocaña, Cristina Nieto-Jiménez, Atanasio Pandiella

**Affiliations:** ^1^ Unidad de Investigación Traslacional, Hospital Universitario de Albacete, Universidad de Castilla La Mancha, Albacete, Spain; ^2^ Instituto de Biología Molecular y Celular del Cáncer and CIBERONC, CSIC-Universidad de Salamanca, Salamanca, Spain

**Keywords:** breast cancer, BET inhibitors, novel targets

## Abstract

Tumoral cells not only depend on oncogenic abnormalities to maintain its malignant phenotype but on non-oncogenic vulnerabilities. Targeting epigenomics can modify specific cellular functions required for malignant transformation. The Bromodomain (BRD) family mediates their effect by recruiting proteins of the transcription machinery, recognizing acetylated-lysine residues in nucleosomal histones. Bromodomain and extra-terminal (BET) inhibitors have shown to produce growth inhibition in several tumors through the inhibition of the expression of several transcription factors. In this review we will discuss the current knowledge regarding BET inhibitors in breast cancer. Recent data demonstrates their antiproliferative effect in several cancer subtypes, including the triple negative subtype, or when combined with cell signaling inhibitors. We will also describe options for therapeutic combinations or potential mechanisms of resistance, with special emphasis on their future clinical development.

## Role of epigenetic regulators in breast cancer

Breast cancer is one of the most prevalent tumors and a leading cause of death in women worldwide [1]. Although early detection and adjuvant treatment have improved survival in early stage breast cancer, disseminated cancer is still an incurable disease where novel therapies have shown limited efficacy [2]. In addition, the fact that most breast cancer patients progress after a given period of treatment reinforce the necessity for the identification of novel therapeutics that can contribute to improve patient outcome.

Breast cancer has been classified in several different subtypes based on molecular and genomic findings [2]. Around 20% of tumors overexpress the transmembrane protein HER2 due to gene amplification [3, 4]. These tumors benefit from therapies against this protein, including monoclonal antibodies like trastuzumab or pertuzumab, or small tyrosine kinase inhibitors like lapatinib or neratinib [5, 6]. Recently targeting tumoral cells with an antibody drug conjugate (ADC) against HER2 has also shown efficacy in the metastatic setting [7]. Expression of hormone receptors (HR), including estrogen and progesterone receptors, is observed in 80% of tumors and strategies to block these receptors or decrease the presence of activating hormones have been used with successful results for a long time [8]. Novel agents designed to block the estrogen receptor have also reached the clinical setting [8, 9]. Finally, breast cancer has been classified by gene expression analyses in several subtypes that mimic the histological classification in some aspects. The HER2 enriched, the luminal and the basal-like subtype mimic tumors overexpressing HER2, HR positive and the triple negative subtype, respectively [8, 10].

**Figure 1:**
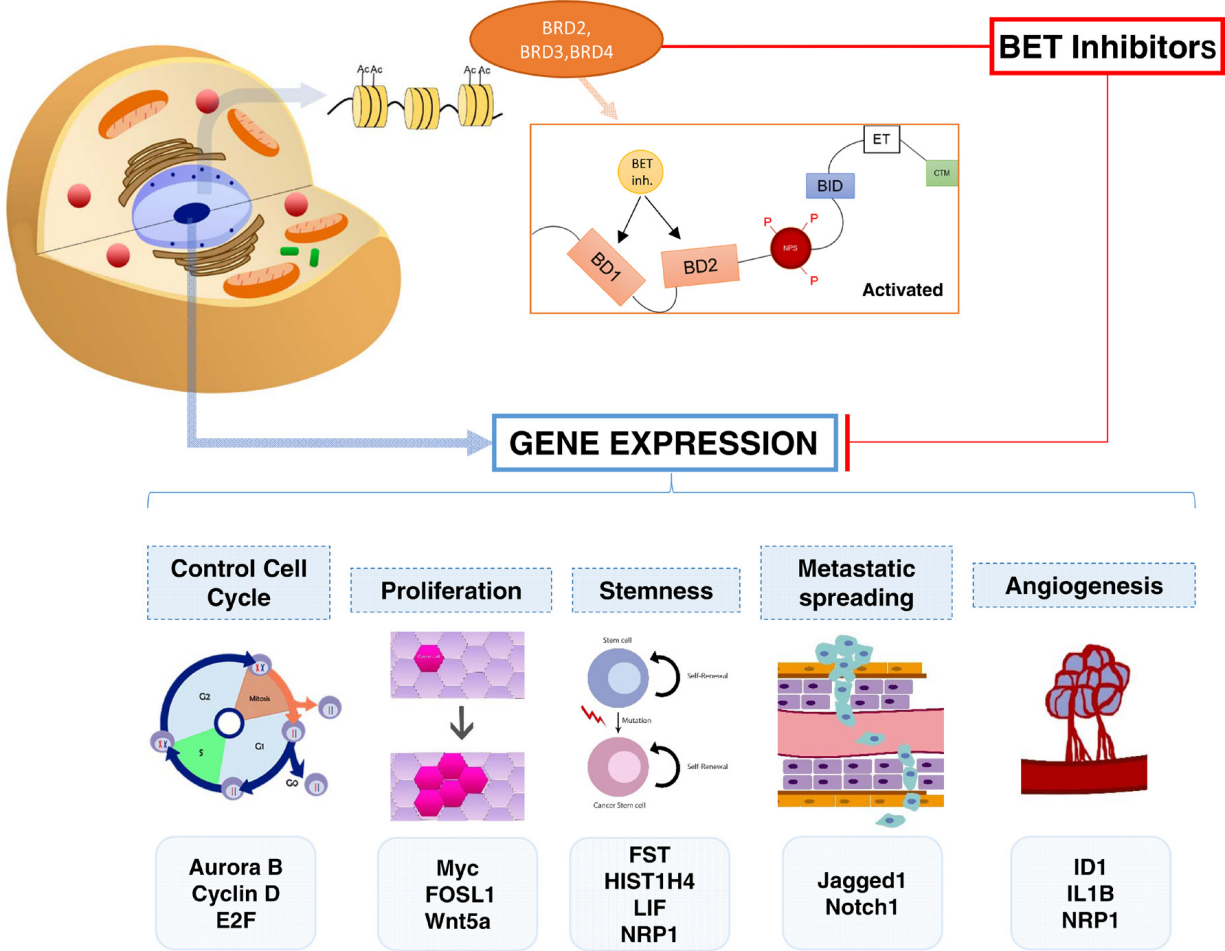
Representation of the mechanisms of action of BET inhibitors and the biological functions inhibited by these compounds The acetylation of lysine residues plays a major role in the regulation of chromatin and the control of its transcription. The extra terminal bromodomains are the main readers of the acetylated lysines producing the transcription of genes related to multiple functions like cell cycle, proliferation, stemness, metastatic spreading and angiogenesis. BET inhibitors suppress the transcription of genes including Aurora B, Cyclin D, E2F, MYC, FOSL1 and Wnt5a, among others.

Epigenetic regulators are promising therapeutic targets in hematologic malignancies and solid tumors [11]. For instance, deacetylase inhibitors have shown activity in the clinical setting in some hematological diseases [12]. Deregulation in chromatin modulators and transcription factors (TFs) are common findings in cancer [11, 13, 14]. The bromodomain and extra-terminal (BET) family of proteins is involved in the recruitment of proteins of the transcription machinery to regulate gene expression [13, 15]. To do so, they recognize acetylated-lysine residues in nucleosomal histones [13, 14]. An example is the recruitment of the positive transcription elongation factor (p-TEFb) [16]. There are around 46 bromodomain-containing proteins of which the BET family in mammals is composed of four members: BRD2, BRD3, BRD4 and the testis-specific BRDT. They contain two conserved N-terminal bromodomains (BD1 and BD2), which recognize and bind acetylated lysine residues on histone tails [17] (Figure 1). Inhibition of these proteins has shown antitumoral efficacy in a wide range of solid tumors including breast cancer [11, 18]. It should be mention, that the inhibition of each individual component can have a different effect that the inhibition of all members and sometimes one member can have an opposite function [19]. On the other hand, most available BET inhibitors can inhibit all members although there are currently under development inhibitors of specific members, like MZ1 that induces selective removal of BRD4 [20].

## Antitumor activity and mechanism of action of BET inhibitors in breast cancer

The efficacy of BET inhibitors was mainly explored in hematologic malignancies and pediatric cancers where well known transcription factors play a key role in the oncogenesis of the disease, as is the case of C-MYC in neuroblastomas or leukemia [18, 21]. Later on, the significant antitumor activity of these compounds in other solid tumors like prostate, non-small cell lung cancer or pancreatic cancer was evaluated and reported [22–24]. Indeed, the efficacy of these agents can be more relevant in biological situations like in cancer where chromatin is in a constitutively open situation available for gene transcription.

BET inhibitors have shown activity in different breast cancer subtypes including HER2 positive tumors, HR tumors and triple negative breast cancer (TNBC). For a comprehensive review of BET inhibitors in clinical development we suggest to read the article by Andrieu G et al. [25].

BRD4 activity is necessary for the proliferation of breast and endometrial tumors expressing estrogen receptors as it regulates ERα-induced gene expression by affecting elongation-associated phosphorylation of RNA polymerase II (RNAPII) and histone H2B monoubiquitination [26]. Indeed BET inhibition with JQ1 impaired E2-induced transcriptional activation [27].

In HER2 and HR positive tumors, BET inhibitors have contributed to overcome resistance to currently approved therapies like lapatinib in HER2 positive tumors, or tamoxifen and everolimus in HR positive cancers, among other therapies [28–30]. These data will be described in detailed in the next section of this review.

Given the fact that BET inhibitors can repress the expression of several transcription factors, the mechanism of action of these compounds is heterogeneous affecting several biological functions including: control of cell cycle, proliferation, stemness, metastatic spreading or angiogenesis, among others [31–34]. Of note doses used in some of the studies were extremely high leading to conclusions that have no relation with the biological mechanism of action [29]. Therefore this data cannot be considered as a good example of how BET inhibitors suppress certain transcription factors and cause arrest of the cell cycle, unless the goal is to generate chemoresistance clones.

Several studies have described the function of Bromodomains (BRD) and particularly BRD2 and BRD4, in cell cycle regulation [35]. Indeed, experience from our group and others shows that treatment with BET inhibitors affects proliferation by inducing cell cycle arrest at G1 with an increase of p27 [33, 34, 36]. Inhibition of proliferation is likely mediated by a reduction of genes involved in cell division [28] and particularly of those associated with the mitotic process like Aurora kinases A and B, Polo-like kinases (PLKs) or TTK protein kinases [33, 36].

Inhibitors of BET proteins reduce cell migration and invasion, and recent data suggests that this mechanism is mediated by inhibition of the Jagged1/Notch1 signaling pathway through modulation of BRD4 [31]. In addition, BET inhibition reduces angiogenesis in xenografted tumors by targeting the hypoxic response [32]. BET inhibition also produce morphologic changes inducing a stem cell phenotype [31, 34, 37]. Interestingly the mechanism of action of BET inhibitors in TNBC is not mainly mediated by the inhibition of MYC or by affecting known pathways linked with this disease like the JAK/STAT pathway [34, 36]. By contrast, other transcription factors affected by the compound have been described like LM04, DEPDC or FOXM1 [33].

Finally, several mechanisms have been reported in relation to resistance to BET inhibitors. The activation of casein kinase II (CK2) has been associated with resistance to BET inhibitors and agents targeting this kinase have shown to overcome such resistance [23]. Similarly, the decrease of the PP2A phosphatase that opposes the CK2 function has been linked with resistance to BET inhibitors [24]. In addition, inhibition of BCL-X and BCL-2, revert the resistance to BET inhibitors [34]. Confirming these findings, novel BET inhibitors with a more potent mechanism of action have shown to be synergistic with agents that induce apoptosis [38]. Of note this has also been observed in other tumors beyond breast cancer, like in T-cell acute lymphoblastic leukemia [39]. Figure 1 summarizes the mechanism of action of BET inhibitors.

## BET inhibitors can overcome resistance to breast cancer therapies

If resistance to a given therapy is mediated by upregulation of specific transcription factors, BET inhibitors can overcome resistance to this specific therapy by reducing their expression. For instance, resistance to everolimus in estrogen receptor positive tumors was mediated by the expression of MYC, and reverted by treatment with BET inhibitors that downregulated MYC expression [29]. Other studies have shown activity of BET inhibitors in HR positive tumors that are resistant to tamoxifen or increasing the effect of the ER degrader fulvestrant [28]. In addition, JQ1 increases the efficacy of everolimus in MCF7 xenograted models [29].

Similarly BET inhibitors can overcome the resistance to PI3K inhibitors [40]. In addition, OTX015, a novel inhibitor, increases the activity of everolimus in some triple negative breast cancer cells [41].

In HER2 positive breast cancer, BET inhibitors have been shown to overcome resistance to lapatinib [30]. Resistance to the anti-HER2 small tyrosine kinase inhibitor lapatinib was mediated by reprograming of the kinome and activation of ErbB2/ErbB3 receptors [30]. BET inhibition reduced the transcription of some of the genes involved in this pathway like ERBB3, IGF1R, DDR1 or MET, among others [30].

## Potential combinations, biomarker discovery and future clinical development

Preclinical data from our group showed that BET inhibitors synergized with chemotherapies used for the treatment of breast cancer including antimitotic agents like taxanes or vinorelbine and DNA damaging compounds like cisplatin and carboplatin [33]. In addition, the combination produced a remarkable induction of apoptosis compared to treatment with each agent given alone [33]. BET inhibitors synergized with agents targeting key kinases involved in the mitotic spindle formation like polo-like kinase 1 [37]. These data opened options for the future clinical development of BET inhibitors in triple negative breast cancer in combination with chemotherapies or PLK1 inhibitors [37].

Similarly BET inhibitors have shown a synergistic interaction with inducers of apoptosis, like BCL-X and BCL-2 inhibitors or with CK2 inhibitors, through the control or relevant phosphatases [34, 38].

Combination of BET inhibitors with immunotherapies like checkpoint inhibitors is an attractive approach. Indeed some articles have suggested that PD-L1 expression is suppressed by treatment with JQ1, and this could be the bases to explore this regimen in several tumors [42].

Also a potent inhibitory effect has been observed with the combination of BET inhibitors and HDAC inhibitors in triple negative and estrogen receptor positive breast cancer cells, mainly through an induction of the ubiquitin-specific protease (USP17) [43]. And finally, the BRD family of proteins has been involved in several mechanisms associated with resistance to cell signaling inhibitors by avoiding the activation of complementary signaling pathways [44]. For instance, inhibition of BRD4 overcomes resistance to MEK inhibitors in triple negative breast cancer [45].

Although not yet explored in breast cancer, in other tumor types like in leukemia the presence of stem cells is associated with resistance to BET inhibitors through an increase of the Wnt/β-catenin pathway [46]. In addition, activation of the AMPK pathway is associated with JQ1-induced autophagy in resistant cells. AMPK promoted JQ1-induced apoptosis in acute myeloide Leukemia stem cells [47].

Table 1 shows the list of BET inhibitors in clinical development. Of note two of them OTX015 and MK-8628 are under evaluation in triple negative breast cancer. Indeed OTX015 has shown a good tolerability profile, with mild side effects like hematologic toxicities, diarrhoea or fatigue, in recently published phase I studies in lymphoma and acute leukemia [48, 49].

**Table 1:** BET inhibitors in clinical development including indication and clinical stage

Status	Reference	Drug	Sponsor	Phase	Condition	Combination
Recruiting	NCT02543879	FT-1101	Forma Therapeutics, Inc.	I	Acute Myeloid Leukemia, Acute Myelogenous Leukemia Myelodysplastic Syndrome	Alone
Recruiting	NCT01949883 NCT02157636 NCT02158858	CPI-0610	Constellation Pharmaceuticals	I	Lymphoma/Multiple Myeloma/Leukemia, Myelocytic, Acute, Myelodysplastic Syndrome (MDS) Myelodysplastic/Myeloproliferative Neoplasm, Unclassifiable Myelofibrosis	Alone
Recruiting	NCT01943851 NCT01587703 NCT02630251	GSK525762 GSK2820151	GlaxoSmithKline	I	Cancer/Carcinoma, Midline/Cancer	Alone
Recruiting	NCT02711956 NCT02705469	ZEN003694	Zenith Epigenetics	I	Metastatic Castration-Resistant Prostate Cancer/Metastatic Castration-Resistant Prostate Cancer	Enzalutamide/alone
Active, not recruiting	NCT02259114	OTX015	Oncoethix GmbH	I	NUT Midline Carcinoma, Triple Negative Breast Cancer Non-small Cell Lung Cancer With Rearranged ALK Gene/Fusion Protein or KRAS Mutation, Castrate-resistant Prostate Cancer (CRPC), Pancreatic Ductal Adenocarcinoma	OTX015/MK-8628
Terminated/Withdrawn	NCT02296476 NCT02303782	OTX015	Oncoethix GmbH	I/II	Glioblastoma Multiforme/ Acute Myeloid Leukemia	Alone/ + Vidaza (azacitidine)
Recruiting	NCT02419417	BMS-986158	Bristol-Myers Squibb	I/II	Multiple Indications Cancer	Alone
Not yet recruiting	NCT02986919	CPI-0610	University of Texas Southwestern Medical Center	II	Peripheral Nerve Tumors	Alone
Active, not recruiting	NCT02698189 NCT02698176	MK-8628	Merck Sharp & Dohme Corp.	I	AML Including AML de Novo and AML Secondary to MDS DLBC/ NUT Midline Carcinoma (NMC), Triple Negative Breast Cancer (TNBC), Non-small Cell Lung Cancer (NSCLC), Castration-resistant Prostate Cancer (CRPC)	Alone
Recruiting	NCT02308761 NCT01987362	TEN-010	Hoffmann-La Roche	I	Myelodysplastic Syndromes, Acute Myeloid Leukemia/ Solid Tumors, Advanced Solid Tumors	Alone
Terminated	NCT02369029	BAY 1238097	Bayer	I	Neoplasms	Alone
Recruiting	NCT02431260 NCT02711137	INCB054329 INCB057643	Incyte Corporation	I/II	Advanced Cancer	Alone
Recruiting/Not yet recruiting	NCT03068351	RO6870810	Hoffmann-La Roche	I	Multiple Myeloma	Alone / Daratumumab
Recruiting	NCT03059147	SF1126	SignalRX Pharmaceuticals, Inc.	I	Advanced Hepatocellular Carcinoma	Alone
Recruiting/Not yet recruiting	NCT02391480	ABBV-075	AbbVie	I	Advanced Cancer, Breast Cancer, Non-Small Cell Lung Cancer (NSCLC), Acute Myeloidm Leukemia, Multiple Myeloma, Prostate Cancer, Small Cell Lung Cance, Non-Hodgkins Lymphoma	Alone / Venetoclax

When observing the clinical development of these compounds, it is interesting to see that there are multiple indications and not clear biological rationale for the development of BET inhibitors in some of them, what suggests that this family of compounds are developed as a tolerated but nonselective molecule.

Strategies to augment the efficacy of these compounds include the designing of more potent agents or the identification of potential biomarkers of response. To increase the efficacy an approach is the development of bivalent BET inhibitors that are 100-fold more potent than monovalent antagonist [50]. To identify biomarkers of efficacy, a recent paper suggests potential genes like HEXIM1, SERPINI1, ZCCHC24 and ZMYND8 that could predict activity in a broader range of tumors [51]. However, these data needs to be confirmed in the clinical setting.

## CONCLUSIONS

In summary, BET inhibitors have demonstrated preclinical activity in TNBC alone or in combination, and in some specific breast cancer scenarios mainly associated with resistance to existing therapies. Their effect goes beyond the regulation of oncogenes as they can act on a variety of cellular programs. The identification of patients whose tumors may benefit from these therapies together with studies to optimize the antitumoral properties of these compounds, represent important future challenges.
